# An approach for diagnosis of diarrhea in neonatal piglets based on the core gut microbiota and machine learning

**DOI:** 10.3389/fmicb.2026.1852304

**Published:** 2026-06-05

**Authors:** Shilong Zhao, Siyi Peng, Huihui Li, Guangxin Yang, Xuefeng Gao, Ke Xu, Lijun Shi, Haitao Yu, Shiyan Qiao

**Affiliations:** 1 State Key Laboratory of Animal Nutrition and Feeding, College of Animal Science and Technology, China Agricultural University, Beijing, China; 2 State Key Laboratory of Animal Nutrition and Feeding, Institute of Animal Science, Chinese Academy of Agricultural Sciences, Beijing, China; 3 Liaoning Vica Agriculture and Animal Husbandry Ecological Food Co., Ltd., Liaoning, China; 4 Beijing Jiahua Pig Breeding Co., Ltd, Beijing, China

**Keywords:** biomarker, diarrhea diagnosis, gut microbiota, machine learning, neonatal piglet

## Abstract

Diarrheal diseases, such as yellow dysentery and white dysentery caused by pathogens or viruses, in newborn piglets lead to substantial economic losses in the swine industry worldwide. Gut microbiota dysbiosis is frequently observed in diarrheic piglets and is thought to play a role in disease pathogenesis, although causal relationships remain to be established. However, developing reliable microbiome-based diagnostic tools still poses a significant challenge. This study aimed to develop a diagnostic model for piglet diarrhea by integrating core microbiota analysis with machine learning. Fecal samples from diarrheic and healthy piglets were subjected to metagenomic sequencing to characterize archaeal, bacterial, and fungal communities. We identified diarrhea-associated bacterial biomarkers via LEfSe, DESeq2, and microbial cooccurrence network analysis. These microbial features were used to construct and compare multiple machine learning classifiers. Our results revealed significant disparities in the structure and diversity of the gut microbiota between diarrheic and healthy piglets, with the bacterial community showing the most notable changes. Among the models developed, the decision tree classifier based on bacterial genus-level features achieved the highest prediction accuracy of 91.18%. Furthermore, a simplified model utilizing a panel of 18 core bacterial genera also demonstrated high efficacy, with a support vector machine model achieving 88.24% accuracy. In independent validation using our internal dataset, the random forest model exhibited the best generalizability and stability. This study establishes a robust, microbiota-based diagnostic model for diarrhea in neonatal piglets, highlighting the potential of machine learning in leveraging microbiome data for disease classification and health management in livestock production.

## 1 Introduction

Diarrheal diseases caused by pathogenic microorganisms or viral infections lead to increased neonatal piglet mortality, which is an economically devastating issue in commercial swine production and adversely affects animal welfare, growth performance, and farm profitability. The gut microbiota, a complex ecosystem integral to host health, plays a pivotal role in nutrient metabolism, immune system development, and protection against pathogens (Gu et al., 2025; Schluter et al., 2020; Wu et al., 2023). Consequently, maintaining a stable gut microbial community is essential for the health and development of piglets from newborn to weaned.

Numerous studies have linked diarrhea to profound disruptions in this microbial ecosystem. For example, infection with enterotoxigenic *Escherichia coli* (ETEC), a primary causative agent, significantly alters the structure and function of the gut microbiota, which in turn modulates intestinal immune responses (Bin et al., 2018). ETEC challenge has been shown to reduce the number of goblet cells, increase populations of sulfate-reducing bacteria and enterococci, and paradoxically increase alpha diversity while depleting beneficial bacteria (Xu et al., 2023). Other studies reported microbial shifts in diarrheic piglets characterized by increased abundances of *Prevotella*, *Sutterella*, *Campylobacter*, and *Clostridiaceae* and a decrease in Firmicutes, suggesting a strong link between this dysbiosis and impaired nutrient absorption and diarrhea (Yang et al., 2017). In addition to ETEC, the cocolonization of non-ETEC *E. coli* and *Enterococcus* presents another pathway to diarrhea (Jonach et al., 2014). Similar microbial disruptions, including an expansion of *Shigella-Escherichia* and a reduction in short-chain fatty acid-producing bacteria, are observed in viral enteritis, such as porcine epidemic diarrhea virus (PEDV) infection, underscoring the strong association between microbiota alterations and diarrhea pathogenesis. (Huang et al., 2019). In weaned piglets, Fu et al. recently demonstrated that bacterial infections (e.g., ETEC and Clostridium perfringens) activate the NLRP3 inflammasome-pyroptosis pathway, leading to intestinal barrier disruption and increased diarrhea incidence, accompanied by reduced microbial diversity, an elevated Firmicutes/Bacteroidetes ratio, and enrichment of pathogens (Fu et al., 2025). These findings collectively underscore the strong association between microbiota alterations, inflammatory responses, and diarrheal pathogenesis.

Despite these associations, the precise host–microbiome interactions and drivers of dysbiosis remain elusive. A significant challenge lies in distilling actionable biomarkers from complex microbiome datasets and translating them into reliable diagnostic tools. The emergence of machine learning (ML) offers a powerful solution for deciphering high-dimensional biological data. As a subset of artificial intelligence, ML algorithms can automatically learn patterns from data and improve through experience, outperforming tasks such as classification and regression (Li et al., 2021). Recently, ML models trained on gut microbiome data have shown exceptional performance in identifying disease biomarkers and enabling diagnostic prediction in human medicine (Li et al., 2025). Machine learning has proven effective in microbiome-based disease classification. For example, a recent study using gut viral metagenomic data achieved high accuracy in discriminating diarrheic from healthy neonatal piglets via a random forest model (Wang et al., 2023). This suggests that similar approaches could be applied to bacterial microbiome data, which are more routinely collected and biologically better characterized in the context of host nutrition and immunity. In industrial pig production, machine learning has already demonstrated its feasibility for disease prediction. Using a random-forest classifier trained on pre-weaning faucal 16S rRNA data, recently predicted post-weaning diarrhea risk with high accuracy (AUC = 0.94 and 0.82 in two independent farms), confirming that ML can effectively distinguish susceptible from resilient piglets based on pre-illness gut microbial features (Zhu et al., 2024). These findings provide a strong methodological precedent for our work and support the approach of cross-cohort validation. However, to our knowledge, no machine learning-based diagnostic tool has been developed specifically for pre-weaning piglet diarrhea using multi-cohort shotgun metagenomic data. We propose that integrating ML with comprehensive microbiome analysis can systematically reveal the dynamic interactions between the host and its microbiota in piglets. This study aimed to systematically compare the gut microbial characteristics of diarrheic piglets by integrating public and self-collected metagenomic data and to construct diagnostic models via multiple machine learning algorithms. We specifically evaluated the generalizability of the models across different cohorts and identified a panel of core bacterial biomarkers, with the goal of providing a reliable classification tool, while acknowledging that the identified microbial biomarkers reflect associations rather than causal mechanisms. We hypothesized that a machine learning classifier trained on publicly available metagenomic data from multiple studies, and validated on an independent self-collected cohort, would distinguish diarrheic from healthy piglets based on gut bacterial composition. Specifically, we predicted that a model using genus-level features would achieve higher classification accuracy than simple univariate indices (such as the Firmicutes-to-Bacteroidetes ratio) and would identify a reproducible set of core bacterial genera associated with diarrheic status.

## 2 Materials and methods

### 2.1 Animal ethics

All the experimental procedures applied in this study were reviewed and approved by the Institutional Animal Care and Use Committee of China Agricultural University (Beijing, CAU20150925-2, AW32013202-1-1).

### 2.2 Experimental data and sample collection

#### 2.2.1 Public metagenomic data acquisition and screening

This study incorporated published metagenomic datasets of piglet feces (Gaio et al., 2021; Guitart-Matas et al., 2024; Tao et al., 2022; Wang et al., 2023) for model training and biomarker discovery. Inclusion criteria were: (1) piglets at approximately 20 days of age or explicitly labeled as pre-weaning piglets; (2) fecal or rectal samples capable of representing the gut microbial composition; (3) individually traceable health/diarrhea phenotypes; and (4) availability of raw shotgun reads that could be subjected to a unified reanalysis pipeline. Exclusion criteria comprised: unknown age or phenotype, non-intestinal source, only 16S amplicon data or processed matrices provided, duplicate samples, and samples with insufficient valid microbial sequences after quality control. For studies containing intervention arms, only subgroups with unambiguous phenotype and treatment information that did not confound the health/diarrhea grouping were retained. All public raw reads were reprocessed through a uniform workflow encompassing quality control, host read filtration, taxonomic annotation, and abundance quantification to eliminate analytical bias. After reprocessing, a compatibility assessment was performed by integrating metadata consistency, sequencing quality, annotation resolution, and community structure profiles (α-diversity, Bray–Curtis distance, and UMAP distribution); samples exhibiting aberrant quality or extreme distributional patterns were excluded. Public data were used exclusively for model training and feature selection; the in-house sequencing data served as an independent validation set and were at no stage involved in feature selection or parameter optimization. The detailed metadata (breed, age, weaning status, health status, and source reference) for each public sample are summarized in Supplementary Table 1.

#### 2.2.2 In-house experimental sample collection and data acquisition

In-house samples were collected from Duroc × Landrace × Large White three-way crossbred piglets at approximately 20 days of age, raised under a single production system (Beijing Jiahua Swine Breeding Company). All piglets were housed with their sows in identical farrowing pens (1.8 × 2.2 m) on slatted plastic floors. The pens were maintained at 28–30°C using overhead infrared lamps during the first week, gradually decreased to 24–26°C by day 20. A 12h light/dark cycle was applied, with automatic ventilation to maintain air quality. Piglets had unrestricted access to suckle their sows; no creep feed was provided. Diarrhea was determined by the presence of loose or unformed feces and perianal soiling, whereas healthy controls had formed feces, normal demeanor, and no recent history of diarrhea. The study period lasted from birth (day 0) to the day of sample collection (day 20). Inclusion criteria were: age within the designated study phase, complete phenotype and sampling information, no administration of antibiotics, antidiarrheal agents, probiotics, or other microbiome-disrupting interventions prior to sampling, and DNA quality and sequencing data meeting analytical requirements. Exclusion criteria were: ambiguous phenotype, missing essential information, coexistence of severe comorbidities, receipt of untraceable interventions, sample contamination or repeated freeze–thaw cycles, substandard DNA, or insufficient sequencing depth. Sampling focused on rectal feces, supplemented in part by freshly voided feces; for diarrheic piglets, rectal samples were prioritized to minimize perturbation of the microbial community by environmental contact. Samples were placed in sterile cryovials, transported under cold-chain conditions, and subsequently subjected to DNA extraction and metagenomic sequencing. All animal-related procedures were performed in strict accordance with the China Agricultural University Institutional Animal Care and Use Committee (Beijing, China, AW32013202-1-1).

#### 2.2.3 Fecal sample collection

Fresh fecal samples were collected as soon as possible after defecation. To avoid environmental contamination, the perineal area of each piglet was cleaned with 70% ethanol and dried with sterile gauze before sampling. Using sterile gloves, approximately 2–3 g of freshly voided feces was directly taken from the rectum and immediately transferred into a sterile 5 mL cryotube. Care was taken to avoid touching the tube exterior with the gloves. The tubes were sealed, labeled with collection date and sample ID, placed in a cool box with ice packs, and transported to the laboratory within 24 h. All procedures were completed within 1 min per piglet to minimize environmental exposure.

##### 2.2.4 Intestinal content collection

Piglets were euthanized by intravenous injection of sodium pentobarbital (150 mg/kg body weight) in accordance with the China Agricultural University IACUC protocol (approval number AW32013202-1-1). After confirming the absence of reflexes, the abdominal cavity was opened under sterile conditions. The intestinal contents were collected via sterile forceps or a scalpel. Approximately 1–3 g of intestinal content per piglet was placed into a sterile cryotube. The tubes were labeled with the collection date and sample ID, stored in a cool box, and transported to the laboratory within 24 h.

### 2.3 Metagenomic sequencing and bioinformatics analysis

Both fecal and intestinal content samples were subjected to the same DNA extraction protocol. Fecal samples were used as the primary material for model development because they are non-invasive and practical for farm use; intestinal content samples were collected as a reference to validate that the fecal microbiome is representative of the gut luminal community.

DNA extraction was performed as follows: Total genomic DNA was extracted from the fecal samples, and paired-end libraries were constructed and sequenced on an Illumina NovaSeq platform (Illumina Inc., San Diego, CA, United States) at Majorbio Bio-Pharm Technology Co., Ltd. (Shanghai, China). Bioinformatic analysis was performed on the Majorbio Cloud Platform.^1^ Briefly, the raw reads were quality controlled, and the host DNA was removed. Metagenomic assembly was carried out, followed by gene prediction, construction of a nonredundant gene catalog, and gene abundance calculation. Taxonomic annotation was performed against the NR database.

### 2.4 Data analysis

Before downstream statistical analysis, taxonomic abundance profiles from public datasets and self-collected samples were processed using the same bioinformatics pipeline to generate comparable abundance matrices for archaea, bacteria, and fungi. To further reduce potential technical variation among different studies and sequencing batches, batch-effect correction was performed using the ComBat method implemented in the “sva” package in R. The data source or study cohort was specified as the batch variable. Diarrhea status was included in the model matrix as a biological covariate so that disease-associated microbial variation was retained during batch correction. The batch-corrected abundance matrices were used for subsequent diversity analysis, community structure comparison, biomarker screening, and machine learning model construction.

The Chao1 (richness) and Shannon (diversity) indices of the archaeal, bacterial, and fungal communities were calculated via the “vegan” package in R (v4.3.3). The Wilcoxon rank-sum test (the “wilcox.test” function of the R base package) method was used to compare the differences between diarrhea and healthy samples and to evaluate the effect of diarrhea on the alpha diversity of the intestinal flora in piglets. Uniform manifold approximation and projection (UMAP) was performed using the “umap” package in R to visualize differences in microbial community structure between diarrheic and healthy piglets. The input data consisted of the processed and batch-corrected taxonomic abundance matrices, with samples as rows and microbial taxa as columns. UMAP was performed separately for archaeal, bacterial, and fungal communities. The parameters were set as follows: n_neighbors = 15, min_dist = 0.1, metric = “euclidean,” and random_state = 123 to ensure reproducibility. The effects of diarrhea on the composition and structure of the intestinal flora in piglets were evaluated via the Adonis test method, which is based on the Bray–Curtis distance (R language “vegan” package). Permutational multivariate analysis of variance (PERMANOVA) was performed using the adonis2 function in the vegan R package with 999 permutations. The R^2^ value (also called the coefficient of determination) indicates the proportion of variance in the Bray-Curtis distance matrix that is explained by the grouping variable (e.g., diarrheic vs. healthy status). Higher R^2^ values denote stronger separation between groups.

To assess stable associations among the differential genera, a co-occurrence network was constructed separately for healthy and diarrheic groups. Prior to analysis, the relative abundance matrix was transformed using centered log-ratio (CLR) to account for compositional data constraints. Pearson correlations were then computed, and permutation testing (1,000 permutations, sample-label shuffling) was applied to retain only edges with FDR-corrected *p* < 0.05. Additionally, bootstrap stability analysis (1,000 iterations, each sampling 80% of the samples) was performed, and only edges with a stability frequency ≥ 80% were considered robust. Edges that showed consistent correlation direction in both groups were defined as stable.

### 2.5 Feature selection criteria

Bacterial genera present in at least 70% of the public training set samples were retained as candidates. Differential abundance between diarrheic and healthy piglets was assessed using two complementary methods: (i) LEfSe (Linear discriminant analysis Effect Size) with an LDA score threshold > 2.5 and *p* < 0.01 (Wilcoxon test), and (ii) DESeq2 (negative binomial Wald test) with thresholds of |log2 fold change| > 1.5 and false discovery rate (FDR) < 0.01. The intersection of genera identified by both methods was considered as robust differential genera. To assess stable associations among the differentially abundant genera identified by the above criteria, guild analysis (co-occurrence network) was performed separately for healthy and diarrheic groups. Pearson correlations were calculated for all possible genus pairs, and significance was assessed within each group separately (Supplementary Figure 4).

### 2.6 Definition of derived variables

The Firmicutes-to-Bacteroidetes (F/B) ratio was calculated for each sample as the relative abundance of the phylum Firmicutes divided by the relative abundance of the phylum Bacteroidota. The diarrhea index was defined as the relative abundance of Bacteroidota minus the relative abundance of Firmicutes. Both indices served as univariate benchmark classifiers, and their predictive performance is reported in Supplementary Results S3.

### 2.7 Machine learning model establishment

A supervised binary classification framework was used to distinguish diarrheic piglets from healthy piglets. The target variable was diarrhea status, coded as diarrheic or healthy. Candidate predictors were microbial taxonomic abundance features derived from metagenomic profiling at different taxonomic levels, including phylum, class, order, family, genus, and species. For the simplified biomarker-based models, the predictors were restricted to the selected core bacterial genera.

The development, validation, and reporting of the machine learning prediction models were revised with reference to the TRIPOD+AI reporting guidance (Collins et al., 2024) and the Consolidated Reporting of Machine Learning Studies (CREMLS) checklist (El Emam et al., 2024). Relevant items from these frameworks were used to improve the transparency of reporting, including the definition of the prediction task, target outcome, candidate predictors, data sources, eligibility criteria, preprocessing procedures, batch-effect correction, model development strategy, internal resampling procedure, independent validation strategy, and model performance metrics. Briefly, the overall analytical pipeline is summarized in Supplementary Figure 3.

Before model construction, microbial abundance tables were preprocessed using a consistent procedure. Samples with incomplete phenotype information or insufficient sequencing quality were removed. Low-abundance and low-prevalence taxa were filtered to reduce sparsity, and the remaining abundance values were normalized to relative abundance and transformed to reduce skewness. Although all datasets were processed using the same bioinformatics pipeline, potential batch effects caused by different data sources were further corrected using the ComBat method. In the ComBat model, data source or study cohort was specified as the batch variable, and diarrhea status was included as a biological covariate to avoid removing disease-associated microbial variation. The processed and batch-corrected abundance matrices were used for model training, testing, and independent validation.

To address class imbalance, random oversampling of the minority class was performed within the training subset of each fold during 10-fold cross-validation. The held-out validation fold was not oversampled and retained its original class distribution. No synthetic oversampling methods, such as SMOTE, were used. Model performance was evaluated on the original validation folds using accuracy, sensitivity, specificity, and AUC.

The public datasets were used as the training set, whereas the self-collected samples were retained as independent validation datasets for external model evaluation. To assess model generalizability across different sample backgrounds, the self-collected data were evaluated using three validation strategies: a combined validation set including all self-collected samples (*n* = 68), a Jie validation set including samples that had undergone a specific intervention (*n* = 34), and a Kong validation set consisting of samples from the blank control group (*n* = 34). By comparing model performance across these three validation sets, we sought to select an independent dataset that most objectively reflected the discriminatory ability of the models for final evaluation and comparison.

A variety of supervised classification algorithms were implemented and compared, including random forest (RF), support vector machine (SVM), logistic regression (LR), least absolute shrinkage and selection operator logistic regression (LASSO), naive Bayes (Bayes), k-nearest neighbors (KNN), decision tree (DT), bootstrap aggregating (bagging), gradient boosting (boosting), artificial neural network (ANN), and conditional inference tree (CIT). These models were implemented using the corresponding R packages: “randomForest” for RF, “e1071” for SVM and naive Bayes, “stats” for LR, “glmnet” for LASSO, “kknn” for KNN, “rpart” for DT, “ipred” for bagging, “caret” for boosting, “nnet” for ANN, and “party” for CIT.

For each algorithm, hyperparameters were optimized using 10-fold cross-validation on the training set with a grid search strategy, with prediction accuracy as the optimization metric. The performance of the final optimized models was evaluated on the independent validation datasets using accuracy, sensitivity, specificity, and the area under the receiver operating characteristic curve (AUC), which was calculated using the “pROC” package.

## 3 Results

### 3.1 Differences in gut microbiota diversity and structure between diarrheic piglets and healthy piglets

**FIGURE 1:**
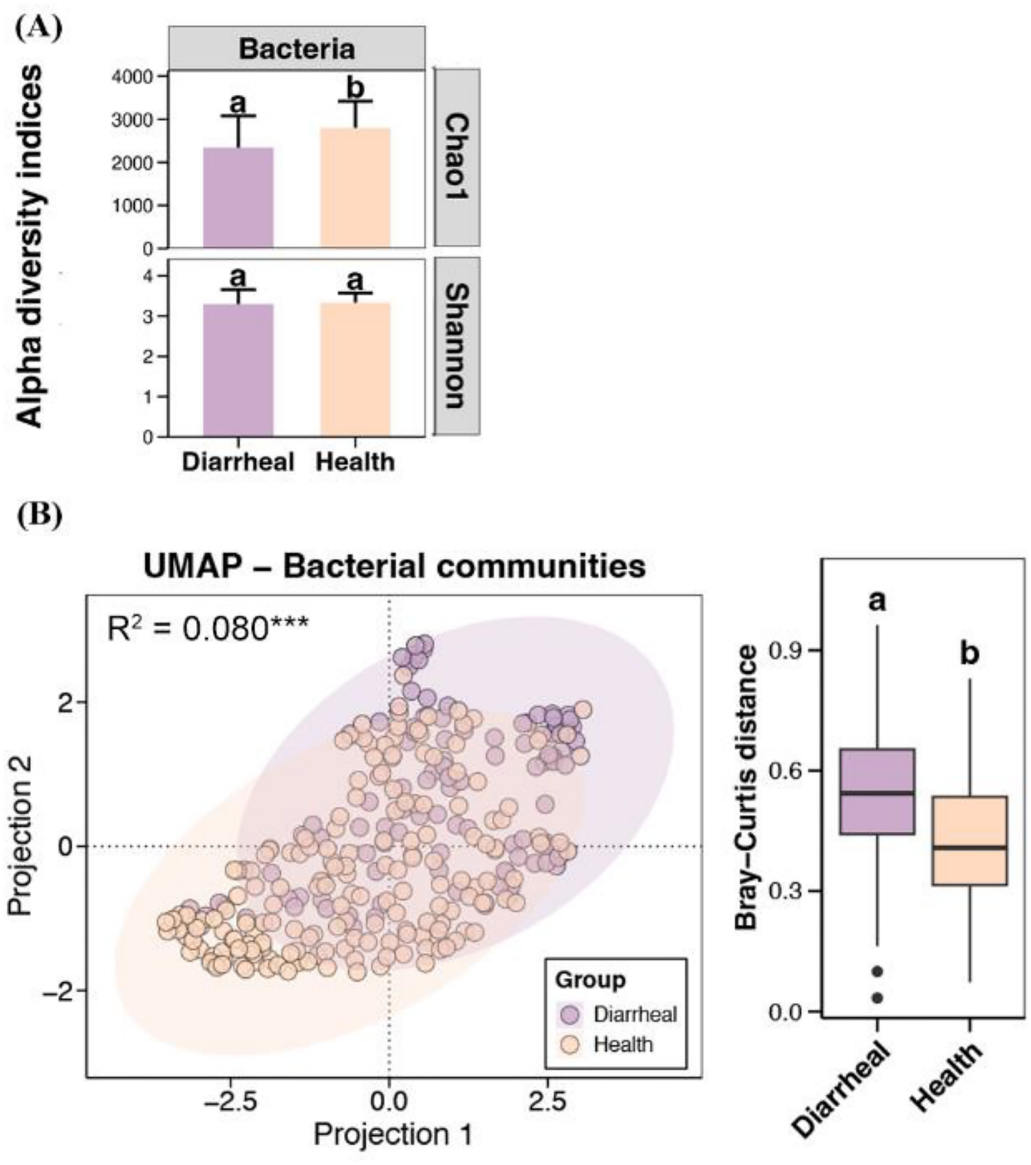
Comparison of the diversity and composition of the intestinal flora between diarrheic and healthy piglets. **(A)** Comparison of α -diversity of gut bacteria between the diarrhea group and the healthy group of piglets (Wilcoxon rank sum test). The vertical coordinates are respectively the Chao1 index (species richness) and the Shannon index (species diversity); Different letters indicated significant differences between groups (*p* < 0.05). **(B)** Based on the Bray-Curtis distance, UMAP was used for dimensionality reduction to demonstrate the β diversity of the gut microbiota, and the degree of community structure dispersion of the two groups of samples was compared. ***Indicates that there is a statistically significant difference among the groups, with a significance level of *p* < 0.001.

To investigate the impact of diarrhea on gut microecology, we calculated the Chao1 and Shannon indices for archaeal, bacterial, and fungal communities from public data and performed Wilcoxon rank-sum tests. Archaeal and fungal community analyses (α/β diversity, taxonomic composition, and machine learning performance) are provided in Supplementary Results 1, as they showed limited contribution to the diagnostic model. The results revealed that bacterial richness (Chao1 index) was significantly lower in diarrheic piglets than in healthy individuals, although no significant difference was found in bacterial diversity (Shannon index) (Figure 1A).

Further dimensionality reduction via uniform manifold approximation and projection (UMAP), combined with Adonis tests based on Bray–Curtis distance, was used to assess the impact of diarrhea on the overall community composition. Bacterial community composition differed significantly between diarrheic and healthy piglets (Adonis *R*^2^ = 0.080, *p* < 0.05; Figure 1B). Archaeal and fungal analyses are provided in Supplementary Results 1. By comparing the dispersion of community structures via the Wilcoxon rank-sum test, we found that the overall variation was significantly greater in the diarrheic samples than in the healthy samples for all three domains (archaea, bacteria, and fungi), suggesting that diarrhea accompanies a more unstable gut microbiota structure.

### 3.2 Comparison of the gut community composition between diarrheic piglets and healthy piglets

**FIGURE 2:**
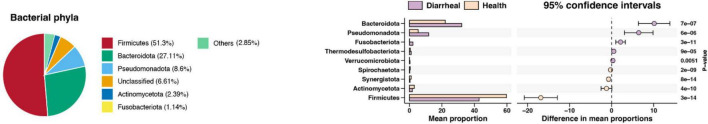
Comparison of gate-level dominant groups of intestinal bacteria in piglets of the diarrhea group and the healthy group (Wilcoxon rank sum test, *p* < 0.05). Phyla with relative abundance < 1% are grouped into “Others.”

In terms of bacterial composition, Firmicutes and Bacteroidota were the dominant phyla, collectively constituting more than 75% of the bacterial community. Wilcoxon rank-sum tests indicated that diarrhea significantly increased the relative abundance of Bacteroidota but significantly decreased that of Firmicutes (*p* < 0.05) (Figure 2). Archaeal and fungal community compositions are described in Supplementary Results 1, respectively.

### 3.3 Comparison of machine learning prediction models

All machine learning analyses were conducted using the ComBat-corrected microbial abundance matrices to minimize potential dataset-specific technical variation. To identify the most robust and representative independent dataset for the final evaluation of our models, we first compared their predictive performance across three distinct test set strategies: the combined set (all self-collected samples, *n* = 68), the Jie set (samples from a specific intervention group, *n* = 34), and the Kong set (samples from the blank control group, *n* = 34). The initial screening revealed that the optimal model and its performance varied depending on the test set used. When the combined test set was used, the boosting model at the family level achieved the highest accuracy of 82.35%. While the Kong test set yielded the highest overall accuracy of 91.18% when a phylum-level decision tree model was used, this result was characterized by strong bias: it achieved perfect accuracy for diarrheic samples (100% sensitivity) but markedly lower accuracy for healthy samples (70% specificity).

In contrast, the Jie test set provided the most balanced and robust performance. The highest accuracy on this set was 88.24%, which was achieved by a genus-level support vector machine model. Crucially, this model demonstrated an excellent balance between identifying diarrheic piglets (83.33% sensitivity) and healthy piglets (100% specificity). Furthermore, the AUC on the Jie test set (0.917) was the highest among all the strategies, indicating superior overall discriminatory power. Therefore, on the basis of its optimal balance of high accuracy, exceptional specificity, and the highest AUC, the Jie test set was selected as the benchmark for all subsequent model evaluations and comparisons.

**FIGURE 3:**
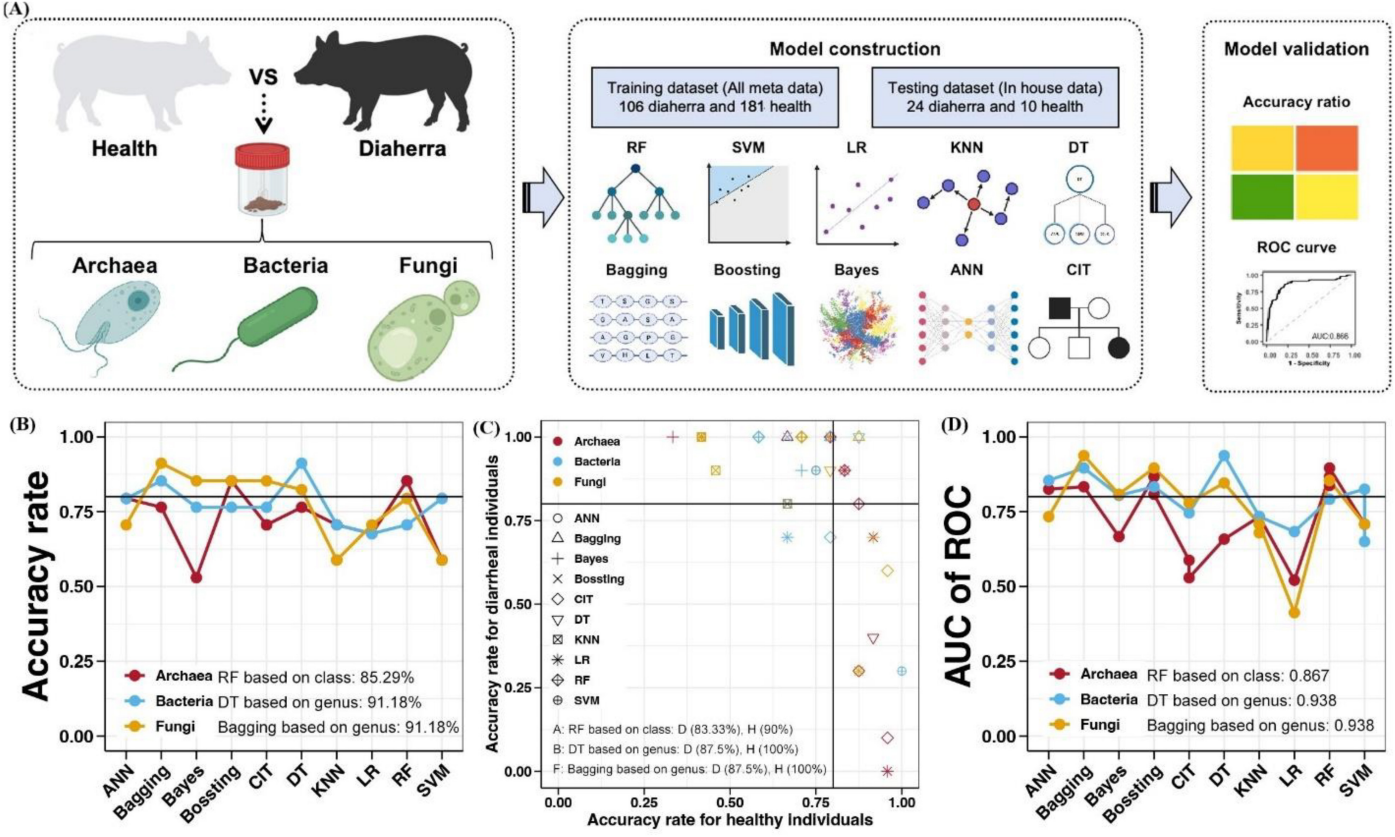
Comparison of machine learning prediction models. **(A)** Summary of the prediction performance of the optimal machine learning models in the domains of archaea, bacteria, and fungi. The horizontal axis represents the model type, and the vertical axis represents the accuracy rate and AUC value. **(B)** Diarrhea prediction performance of the random forest (RF) model at the Archaea level. **(C)** Diarrhea prediction performance of the bacterial genus Horizontal Decision tree (DT) model. **(D)** Diarrhea prediction performance of the horizontal Bagging model of the genus Fungi.

With the Jie test set established as the benchmark, we present the performance of the best-performing models for each microbial domain in detail (Figure 3A). For archeal data, the random forest (RF) model at the class level achieved the highest prediction accuracy of 85.29%, with an accuracy of 87.5% for diarrheic samples and 100% for healthy samples and an AUC of 0.938 (Figure 3B). For the bacterial data, the decision tree (DT) model at the genus level performed best, with a prediction accuracy of 91.18% (Figure 3C). For the fungal data, the Bagging model at the genus level was the most accurate, with an accuracy of 91.18%, an accuracy of 87.5% for the diarrheic samples and 100% for the healthy samples, and an AUC of 0.938 (Figure 3D).

Overall, among all the models and domains evaluated on the Jie test set, the decision tree classifier based on bacterial genus-level features demonstrated the best predictive performance for piglet diarrhea, fungal and archaeal communities showed limited contribution to the diagnostic model performance. Thus, the bacterial community was taken as the primary focus for subsequent biomarker identification and model optimization.

### 3.4 Analysis of bacterial biomarkers

**FIGURE 4:**
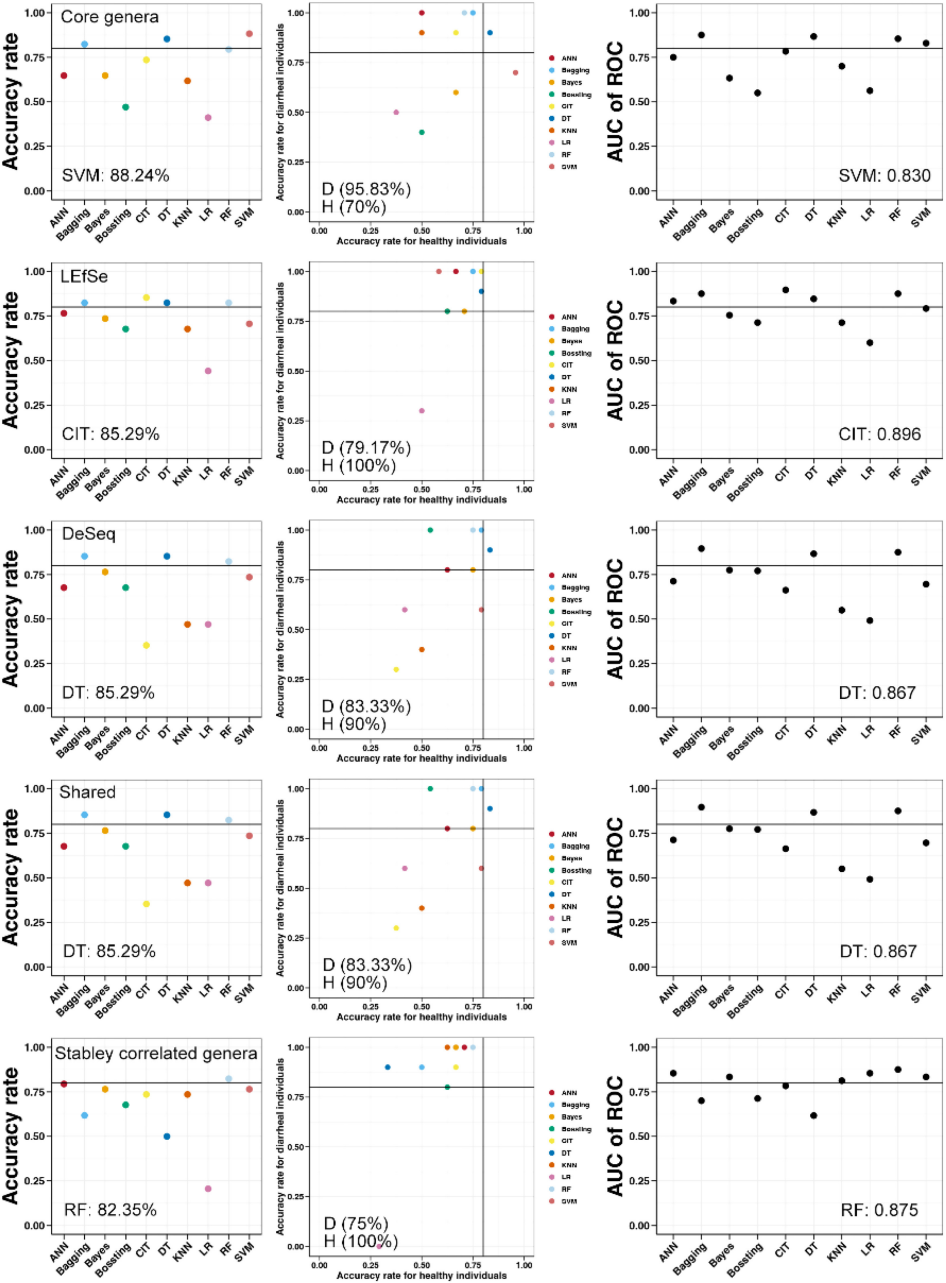
Analysis of bacterial biomarkers. Microbial co-occurrence networks of the 38 differential genera in the healthy and diarrheic groups. Nodes represent bacterial genera, and edges represent significant Pearson correlations. Only 23 genus pairs exhibiting consistent correlation directions across both groups were considered stable, involving 20 genera. After removing 2 unclassified genera, 18 core biomarker genera were finally retained.

The results revealed 112 significantly correlated genera in the healthy group and 62 in the diarrheic group. By comparing the significance and direction of correlations between the two groups, 23 genus pairs with stable, direction-consistent correlations were identified, involving 20 genera. After 2 unclassified or undefined genera were removed, 18 genera were retained for subsequent analysis (Figure 4). For comparison, we also evaluated two simple univariate indices (F/B ratio and a diarrhea index). Both showed substantially lower predictive performance (best accuracy 73.53%) than the machine learning classifiers (detailed results in Supplementary Results 2).

### 3.5 Accuracy of machine learning models on independent validation data

**FIGURE 5:**
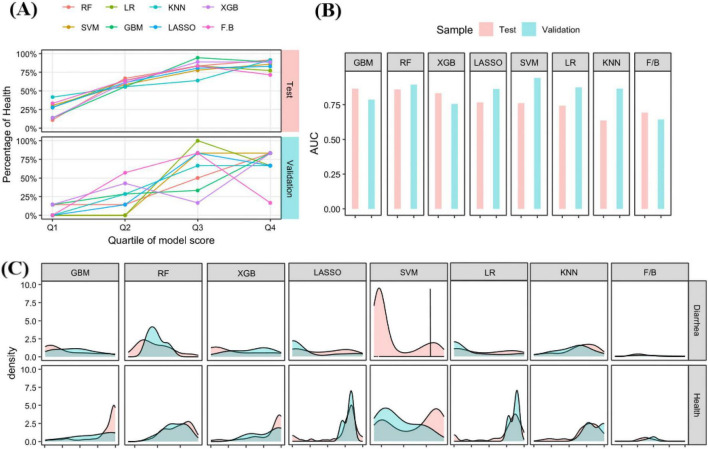
Comparison of the accuracy of different machine learning models for self-test data. **(A)** Comparison of ranking capabilities of each model on public datasets and internal datasets. The model risk score was divided into quartiles from Q1 (high risk) to Q4 (low risk), with the vertical axis representing the proportion of healthy piglets in each quartile. **(B)** Comparison of accuracy and AUC values of each model on the test set (public data) and the validation set (self-built data). **(C)** Score distribution of each model for the diarrhea group/healthy group (public dataset vs. internal dataset).

The performance on the locally generated, independent dataset was crucial for evaluating model robustness and practical utility. As shown in Figure 5B, while the support vector machine (SVM) performed well on the independent test set, its predictive scores differed significantly between the public and internal datasets, indicating poor stability across different data sources (Figure 5C). In addition to SVM, logistic regression (LR), LASSO regression, random forest (RF), and K-nearest neighbors (KNN) also performed reasonably well on the independent dataset. Notably, KNN’s AUC varied considerably in the internal dataset, indicating unstable performance. LR and LASSO regression showed similar results; although their AUCs on the internal dataset were slightly lower than those of the RFs, they were still higher than those obtained on the test set derived from the public data. Among the tree-based ensemble models, the RF model achieved the highest AUC value and the smallest performance discrepancy between the internal and public datasets, indicating high stability. The F/B index performed the worst among all the models, although its values were consistent across datasets, suggesting good stability but poor discriminative power.

Overall, the RF, LR, and LASSO models exhibited a favorable balance of discriminative ability and stability. Specifically, these three models showed similar score distributions for healthy piglets (Figure 5C). Compared with those of the healthy group, the score distributions of the diarrheic group differed markedly between the public and internal datasets. Particularly in the RF model, the scores for the diarrheic group in the internal dataset shifted significantly to the right, whereas the LR and LASSO models showed a slight leftward shift. This discrepancy may stem from differences in the etiological factors causing diarrhea in the public versus the internal datasets.

To further investigate the ranking ability of the models, both public and internal datasets were divided into four quartiles (Q1, Q2, Q3, and Q4) on the basis of risk scores, and the proportion of healthy piglets in each quartile was calculated. As shown in Figure 5A, in the public dataset, the performance differences among the models were small, with only the RF model showing higher predictive accuracy between the lowest (Q4) and highest (Q1) risk groups than the other models did. In the internal dataset, however, the healthy proportions across quartiles varied considerably more among the models. The F/B index performed worst, especially in the lowest-risk quartile (Q4), where more than 75% of the piglets were misclassified as diarrheic, indicating substantial prediction error. Although the LR and LASSO models outperformed the F/B index, the proportion of diarrheic piglets in Q4 was lower than that in Q3, lacking a clear monotonic trend. In contrast, the RF model demonstrated strong ranking capability, with the proportion of healthy piglets progressively increasing as the risk level decreased, indicating reliable predictive power.

Therefore, on the basis of the current data performance, the alert threshold for the RF model can be set at the median value (0.54) of the internal sample scores. Piglets with scores below this threshold had a 93% probability of diarrhea, whereas those above it had a reduced probability of 30%. This threshold can be dynamically adjusted on the basis of actual production needs as the test sample size increases.

## 4 Discussion

### 4.1 Gut microbiota diversity and the F/B ratio in piglets

This study compared gut microbiota diversity between healthy and diarrheic piglets. The results revealed that the overall variation in the archaeal, bacterial, and fungal communities was significantly greater in the diarrheic samples than in the control samples, suggesting that diarrhea may be accompanied by gut microbiota niche restructuring and increased fluctuations. For comparison with conventional indicators, F/B ratio and diarrhea index were evaluated as benchmark methods. The F/B ratio is widely used as a preliminary indicator of intestinal homeostasis in humans. The Firmicutes phylum contains many strains closely associated with gut health. Common lactobacilli, for example, not only increase short-chain fatty acid (e.g., butyrate) production, reducing chronic intestinal inflammation but also inhibit pathogenic microorganisms by competing for resources and producing antimicrobial substances (e.g., lactic acid, hydrogen peroxide, and bacteriocins) (Ahansaz et al., 2023; Xu et al., 2024). Bacteroides primarily ferment dietary or host-derived glycans for energy (Cheng et al., 2022), and their fermentation products can be potentially toxic to the colon (Sears et al., 2014). However, caution is warranted when applying the F/B ratio to piglet health assessment: a lower F/B ratio typically indicates Bacteroidota dominance, potentially leading to reduced SCFA, exacerbated local inflammation, and a weakened mucosal barrier; however, the F/B ratio is not a unidirectional indicator, as excessive Firmicutes expansion might also promote the proliferation of potential pathogens. Therefore, although our study revealed an intuitive association and predictive potential between the F/B ratio and diarrhea, its application in production practice or clinical diagnosis should be combined with other indicators, such as microbial abundance, diversity, and specific functional gene expression levels, to avoid oversimplifying the complexity of the intestinal ecosystem.

### 4.2 Shift from a “single marker” to a “core genera network”

This study identified potential core genera by using three independent methods (LEfSe, DESeq2, network) to enhance stability and reproducibility, rather than outcome-driven selection in healthy and diarrheic groups, and revealed dynamic changes in the microbial network via guild analysis. Core genera often carry out key ecological functions or hold important positions in host–microbe interactions, which are not solely dependent on their abundance. Some core genera play significant roles in intestinal imbalance and inflammatory responses, which are often directly or indirectly linked to diarrhea or gut mucosal damage. For example, enterotoxigenic Escherichia coli (ETEC) can disrupt the balance of intestinal fluid and electrolyte absorption and secretion in piglets; when chloride-ion-driven fluid secretion exceeds the absorption capacity, diarrhea ensues (Bin et al., 2018). *Fusobacterium* is considered a proinflammatory microbe and prognostic biomarker that is capable of suppressing T-cell responses and promoting the expression of inflammatory factors (Wang and Fang, 2023). *Campylobacter* not only causes diarrhea in humans but also has been shown to have increased relative abundance in diarrheic piglets (Yang et al., 2020), although it was only significant at *p* = 0.05 in our study. Most genera showed significant differences in abundance between the two groups (*p* = 0.01), except for *Morganella* and *Actinobacillus*, which were not significantly different (*p* = 0.05), and *Glaesserella*, which was significantly different at *p* = 0.05. Our study also identified some novel or less documented genera, such as *Hominibacterium*, first discovered in human feces by Borhanudin et al. (2022), whose health impacts remain unclear. These microbes may play potential roles in microbial metabolism and host immune regulation, warranting further investigation. Subsequent studies could integrate metatranscriptomics, metabolomics, and culturomics to elucidate the key regulatory roles of these genera in maintaining intestinal homeostasis or triggering diarrhea.

Furthermore, the correlations or co-occurrence networks among core genera reflect “ecological synergy” or “antagonism” within the gut microbiota. The overall weaker correlations in the diarrheic group suggest microbial community destabilization following environmental perturbation or pathogen intrusion. This destabilization, if exacerbated, could lead to more severe clinical symptoms and even impact the growth performance and immune development of piglets later. Therefore, identifying core genera not only deepens the understanding of intestinal ecology but also provides new targets for feed additive or probiotic prevention strategies. Restoring and consolidating the stable network of core genera might achieve a “bacteria-against-bacteria” effect during early intervention in the context of diarrhea risk.

The decision tree classifier (91.18% accuracy) uses a series of binary splits based on the abundance of specific bacterial genera. Its high performance may be attributed to the inherent hierarchical structure of microbial communities: certain genera act as gatekeepers, and their presence or absence strongly predicts diarrheic status. This interpretability is a major advantage for on-farm applications, where understanding the decision rule (“if genus X > threshold then diarrhea”) is as valuable as the prediction itself.

The random forest model showed the smallest performance drop between the public training set and the independent test set (Figure 5C), indicating strong generalizability. This likely reflects the ensemble nature of random forest, which reduces overfitting by averaging many decorrelated decision trees. In contrast, the support vector machine (SVM) and K-nearest neighbors (KNN) were more sensitive to batch effects and dataset heterogeneity, as reflected by their larger performance discrepancies (Figure 5B).

Previous machine learning studies on pig gut microbiota have primarily used 16S rRNA data and focused on weaned piglets. For example, Pirolo et al. applied random forest to 16S data and achieved an AUC of 0.94 in one farm but only 0.82 in a second farm, highlighting the challenge of cross-farm validation (Pirolo et al., 2026). Our decision tree model achieved 91.18% accuracy on a completely independent dataset from a different farm, suggesting that shotgun metagenomic data may provide more robust features than 16S data. However, direct comparisons are limited by differences in age groups (neonatal vs. weaned), sequencing methods, and sample sizes. Future studies should benchmark both approaches on identical cohorts.

### 4.3 Predictive model construction and application prospects

Machine learning algorithms demonstrate significant potential in model accuracy and feature extraction when dealing with complex microbiome data. On the application front, this study validated model performance via independently collected samples, with random forest maintaining high consistency across different data sources. Although the external validation results are positive, the condition linked to the model involves complex and variable factors. Factors such as different feed formulations, rearing environments, and pathogen types may lead to different gut microbial responses. In this study, a systematic comparison of different test sets revealed that the models exhibited distinct predictive preferences depending on the cohort from different treatment backgrounds (Jie vs. Kong). For example, models demonstrated a superior ability to identify healthy individuals within the Jie cohort, whereas they showed greater sensitivity to diarrheic samples in the Kong cohort. This phenomenon underscores the profound modulatory effects of external factors, such as rearing environments and dietary interventions, on the composition of the gut microbiota and its association with disease. This highlights the necessity of considering the applicability and stability of biomarkers across different production systems. Our final model, which demonstrated optimal and stable performance on the relatively balanced Jie test set, provides confidence for its future application. Therefore, future research should incorporate multi-timepoint sampling data from different regions and large-scale farms to establish more comprehensive models tailored to microbial succession patterns at various rearing stages. Additionally, exploring the integration of multimodal data, such as body length, weight, and body temperature, via ensemble and deep learning methods could enhance model generalizability, enabling more accurate disease warning and intervention.

In practical applications, challenges related to detection cost and turnaround time remain. Although metagenomic sequencing is common in research, its routine implementation in farm settings is difficult. Although an independent dataset was used for validation, the relatively small sample size, particularly the limited number of healthy controls, and the single-farm origin of the self-collected dataset may limit the generalizability of our findings. Second, as the models were trained and validated on data from specific rearing environments and breeds, the heterogeneity of public datasets may introduce confounding, their generalizability across different production systems, geographical regions, and swine breeds needs further evaluation. Given that our model relies primarily on microbial community structure data, combining it with lower-cost 16S rRNA sequencing technology for risk assessment and assisting in piglet management may offer greater economic value. Furthermore, optimizing sampling procedures, adopting rapid sequencing technologies, or developing portable detection devices could further reduce costs and shorten turnaround times, facilitating the practical application of the model in farming operations.

## 5 Limitations

Several limitations of this study should be acknowledged.

(1) Sample size and generalizability: The self-collected validation cohort included only 34 piglets (24 diarrheic, 10 healthy) from a single farm. This small sample size, particularly the limited number of healthy controls, may limit the statistical power and generalizability of our findings. Future studies with larger, multi-center cohorts are needed to confirm the robustness of the identified biomarkers.

(2) Public dataset heterogeneity: The public training set comprised four independent studies with varying piglet ages, diets, housing conditions, and diarrhea etiologies (e.g., ETEC, viral infections, stress). Although we used ComBat to correct for batch effects and included “study ID” as a covariate in sensitivity analyses, some unmeasured confounding may remain.

(3) Cross-sectional design and causality: All samples were collected after diarrhea onset; therefore, the observed gut microbiota differences reflect associations with diarrheic status, not causal relationships. Whether the identified core genera are drivers or consequences of diarrhea cannot be determined from our data.

(4) Technical limitations of network analysis : We acknowledge that despite applying CLR transformation and permutation-based filtering, using Pearson correlation on relative abundance data may still be subject to compositional constraints and spurious associations. To partially mitigate this, we adopted a conservative strategy retaining only edges that showed consistent correlation direction in both healthy and diarrheic groups, which prioritizes robustness over sensitivity. Nevertheless, future analyses may benefit from dedicated network inference tools designed specifically for compositional data (e.g., SpiecEasi, SparCC) to further reduce the risk of artifactual associations.

(5) Model validation scope: The random forest model was validated on a single independent farm cohort. Its performance on other production systems, breeds, and geographical regions remains to be tested.

## 6 Conclusion

In conclusion, this study establishes a reliable framework for the diagnosis of diarrhea in neonatal piglets by leveraging gut microbiota signatures and machine learning. The random forest model emerged as the most robust and generalizable classifier upon independent validation, demonstrating strong potential for practical deployment in farming operations. Our findings provide proof-of-concept for microbiota-based diagnosis. Future studies with larger, multi-center cohorts are warranted to further enhance generalizability.

## Data Availability

The raw data generated in this study can be found at: https://www.ncbi.nlm.nih.gov/sra/, PRJNA1416814.

## Footnotes

1
www.majorbio.com

## References

[B1] Ahansaz N. Tarrah A. Pakroo S. Corich V. Giacomini A. (2023). Lactic acid bacteria in dairy foods: Prime sources of antimicrobial compounds. *Fermentation* 9:964. 10.3390/fermentation9110964

[B2] Bin P. Tang Z. Liu S. Chen S. Xia Y. Liu J. et al. (2018). Intestinal microbiota mediates Enterotoxigenic *Escherichia coli*-induced diarrhea in piglets. *BMC Vet. Res.* 14:385. 10.1186/s12917-018-1704-9 30518356 PMC6282381

[B3] Borhanudin N. Yang M. Chaplin A. V. Li J. Wang Q. Dai L. R. et al. (2022). Hominibacterium faecale gen. nov., sp. nov., an anaerobic L-arginine-degrading bacterium isolated from human feces. *Arch. Microbiol.* 205 3334–3139. 10.1007/s00203-022-03365-z 36536120

[B4] Cheng J. Hu J. Geng F. Nie S. (2022). *Bacteroides* utilization for dietary polysaccharides and their beneficial effects on gut health. *Food Sci. Hum. Wellness* 11 1101–1110. 10.1016/j.fshw.2022.04.002

[B5] Collins G. S. Moons K. G. M. Dhiman P. Riley R. D. Beam A. L. Van Calster B. et al. (2024). TRIPOD+AI statement: Updated guidance for reporting clinical prediction models that use regression or machine learning methods. *Bmj* 385:e078378. 10.1136/bmj-2023-078378 38626948 PMC11019967

[B6] El Emam K. Leung T. I. Malin B. Klement W. Eysenbach G. (2024). Consolidated reporting guidelines for prognostic and diagnostic machine learning models (CREMLS). *J. Med. Internet Res.* 26:e52508. 10.2196/52508 38696776 PMC11107416

[B7] Fu J. Jiang Z. Wen C. Su W. Yang M. He H. et al. (2025). Bacterial infection in weaned piglets promotes diarrhea by inducing the NLRP3 inflammasome-pyroptosis pathway. *Sci. China Life Sci.* 68 3021–3036. 10.1007/s11427-024-2728-2 40622657

[B8] Gaio D. DeMaere M. Z. Anantanawat K. Eamens G. J. Liu M. Zingali T. et al. (2021). A large-scale metagenomic survey dataset of the post-weaning piglet gut lumen. *Gigascience* 10:giab039. 10.1093/gigascience/giab039 34080630 PMC8173662

[B9] Gu P. Wei R. Liu R. Yang Q. He Y. Guan J. et al. (2025). Aging-induced alternation in the gut microbiota impairs host antibacterial defense. *Adv. Sci.* 12:e2411008. 10.1002/advs.202411008 39792643 PMC11948050

[B10] Guitart-Matas J. Ballester M. Fraile L. Darwich L. Giler-Baquerizo N. Tarres J. et al. (2024). Gut microbiome and resistome characterization of pigs treated with commonly used post-weaning diarrhea treatments. *Anim. Microbiome* 6:24. 10.1186/s42523-024-00307-6 38702766 PMC11067243

[B11] Huang A. Cai R. Wang Q. Shi L. Li C. Yan H. (2019). Dynamic change of gut microbiota during porcine epidemic diarrhea virus infection in suckling piglets. *Front. Microbiol.* 10:322. 10.3389/fmicb.2019.00322 30858839 PMC6397872

[B12] Jonach B. Boye M. Stockmarr A. Jensen T. K. (2014). Fluorescence in situ hybridization investigation of potentially pathogenic bacteria involved in neonatal porcine diarrhea. *BMC Vet. Res.* 10:68. 10.1186/1746-6148-10-68 24628856 PMC3995547

[B13] Li P. Li M. Chen W. H. (2025). Best practices for developing microbiome-based disease diagnostic classifiers through machine learning. *Gut Microbes* 17:2489074. 10.1080/19490976.2025.2489074 40186338 PMC11980492

[B14] Li Q. Yi Y. Wu Z. Ding T. (2021). Review of gut microbiome analysis prediction models and algorithms. *Microbiol. China* 48 180–196.

[B15] Pirolo M. Sherwani M. K. Espinosa-Gongora C. Eriksen E. Tassinato C. Alberdi A. et al. (2026). Faecal microbiome profiling uncovers putative biomarkers for piglets resilient to post-weaning diarrhoea. *Anim. Microbiome* 8:33. 10.1186/s42523-026-00522-3 41736110 PMC13040825

[B16] Schluter J. Peled J. U. Taylor B. P. Markey K. A. Smith M. Taur Y. et al. (2020). The gut microbiota is associated with immune cell dynamics in humans. *Nature* 588 303–307. 10.1038/s41586-020-2971-8 33239790 PMC7725892

[B17] Sears C. L. Geis A. L. Housseau F. (2014). *Bacteroides* fragilis subverts mucosal biology: From symbiont to colon carcinogenesis. *J. Clin. Invest.* 124 4166–4172. 10.1172/jci72334 25105360 PMC4191034

[B18] Tao S. Zou H. Li J. Wei H. (2022). Landscapes of enteric virome signatures in early-weaned piglets. *Microbiol. Spectr.* 10:e0169822. 10.1128/spectrum.01698-22 35913177 PMC9430488

[B19] Wang N. Fang J. Y. (2023). *Fusobacterium nucleatum*, a key pathogenic factor and microbial biomarker for colorectal cancer. *Trends Microbiol.* 31 159–172. 10.1016/j.tim.2022.08.010 36058786

[B20] Wang Z. Li J. Ma L. Liu X. Wei H. Xiao Y. et al. (2023). Metagenomic sequencing identified specific bacteriophage signature discriminating between healthy and diarrheal neonatal piglets. *Nutrients* 15:1616. 10.3390/nu15071616 37049457 PMC10097093

[B21] Wu D. Xiong W. Ma S. Luo J. Ye H. Huang S. et al. (2023). Konjac flour-mediated gut microbiota alleviates insulin resistance and improves placental angiogenesis of obese sows. *AMB Express* 13:143. 10.1186/s13568-023-01646-4 38087159 PMC10716105

[B22] Xu B. Wang Z. Wang Y. Zhang K. Li J. Zhou L. et al. (2024). Milk-derived Lactobacillus with high production of short-chain fatty acids relieves antibiotic-induced diarrhea in mice. *Food Funct.* 15 5329–5342. 10.1039/d3fo04706g 38625681

[B23] Xu J. Jia Z. Xiao S. Long C. Wang L. (2023). Effects of enterotoxigenic *Escherichia coli* challenge on jejunal morphology and microbial community profiles in weaned crossbred piglets. *Microorganisms* 11:2646. 10.3390/microorganisms11112646 38004658 PMC10672776

[B24] Yang G. Yan Y. Zhang L. Ruan Z. Hu X. Zhang S. et al. (2020). Porcine circovirus type 2 (PCV2) and Campylobacter infection induce diarrhea in piglets: Microbial dysbiosis and intestinal disorder. *Anim. Nutr.* 6 362–371. 10.1016/j.aninu.2020.05.003 33005770 PMC7503086

[B25] Yang Q. Huang X. Zhao S. Sun W. Yan Z. Wang P. et al. (2017). Structure and function of the fecal microbiota in diarrheic neonatal piglets. *Front. Microbiol.* 8:502. 10.3389/fmicb.2017.00502 28392784 PMC5364137

[B26] Zhu J. Sun Y. Ma L. Chen Q. Hu C. Yang H. et al. (2024). Comparative analysis of fecal microbiota between diarrhea and non-diarrhea piglets reveals biomarkers of gut microbiota associated with diarrhea. *Anim. Nutr.* 19 401–410. 10.1016/j.aninu.2024.05.013 39640543 PMC11617881

