# Supplementary material for: An approach for diagnosis of diarrhea in neonatal piglets based on the core gut microbiota and machine learning
Source: Front Microbiol. 2026 Jun 5;17:1852304. doi: 10.3389/fmicb.2026.1852304 (PMC13278932; doi:10.3389/fmicb.2026.1852304)
Supplement: Supplementary file 2 [file Supplementary_file_2.docx]

**Supplementary Results S1: Archaeal and fungal community analyses**


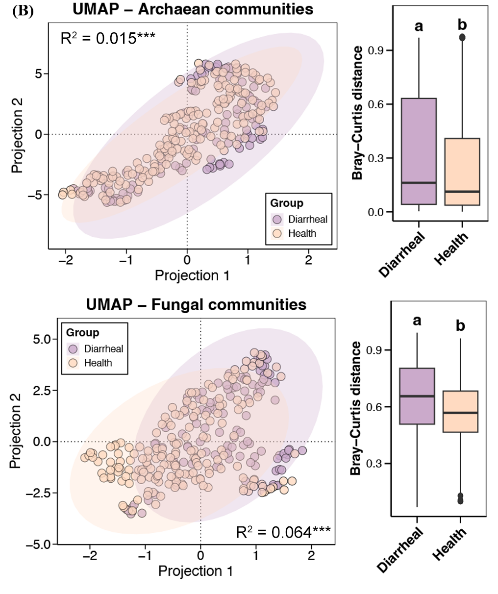

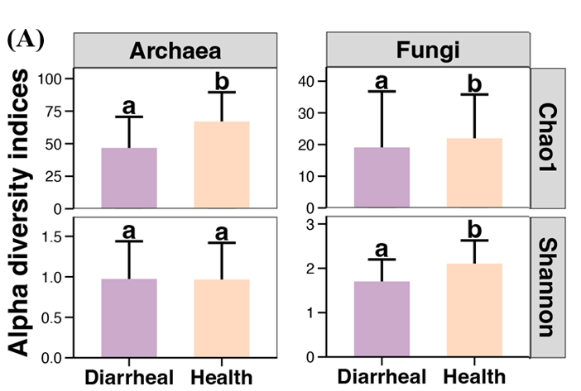
The results revealed that the richness of archaea was significantly lower in diarrheic piglets than in healthy individuals, although no significant difference was found in their species diversity. In addition, both the richness and diversity of fungi were significantly lower in the diarrheic group (Supplementary Figure. S1A). And by using UMAP and Bray‒Curtis distance, the results revealed statistically significant differences in the composition of the archaeal (R² = 0.015) and fungal (R² = 0.064) communities between the two groups (Supplementary Figure. S1B).

**Supplementary Figure. S1**. Comparison of the diversity and composition of the intestinal flora between diarrheic and healthy piglets. (A) Comparison of α -diversity of gut archaea and fungi between the diarrhea group and the healthy group of piglets (Wilcoxon rank sum test). The vertical coordinates are respectively the Chao1 index (species richness) and the Shannon index (species diversity); Different letters indicated significant differences between groups (*p*<0.05); (B) Based on the Bray-Curtis distance, UMAP was used for dimensionality reduction to demonstrate the β diversity of the gut microbiota, and the degree of community structure dispersion of the two groups of samples was compared.

In terms of archaeal composition, the genus *Methanobrevibacter* dominated, accounting for more than 85% of the archaeal community. However, the Wilcoxon rank-sum test revealed no significant difference in abundance between diarrheic and healthy piglets (*p* > 0.05). Further analysis at the species level revealed that diarrhea significantly increased (*p* < 0.05) the relative abundance of three *Methanobrevibacter* species: *Methanobrevibacter sp*., *Methanobrevibacter woesei*, and *Methanobrevibacter oralis* (Supplementary Figure. S2A).

In terms of fungal composition, the communities were largely composed of *Ascomycota* and *Mucoromycota*. However, no taxa at the phylum level presented significantly different abundances between diarrheic and healthy individuals (*p* > 0.05). Further comparison at the species level revealed significant differences (*p* < 0.05) among the four species: diarrhea significantly increased the abundance of *Rhizopus arrhizus* and *Fusarium graminearum* but decreased the abundance of *Ogataea philodendri* and *Fusarium avenaceum* (Supplementary Figure. S2B).


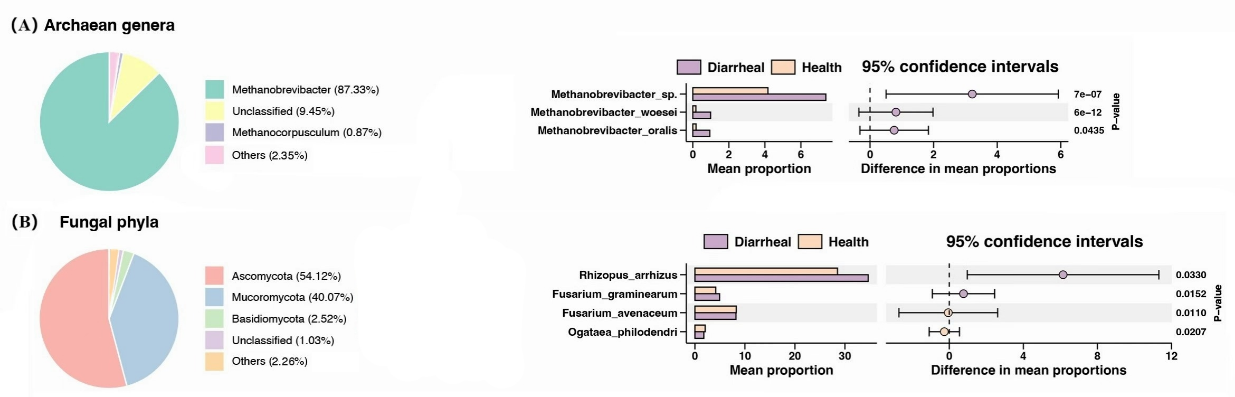


**Supplementary Figure. S2**. Comparison of the intestinal community composition between diarrheic and healthy piglets. (A) Comparison of the dominant groups at the genus level and the differences at the species level of intestinal archaea between the diarrhea group and the healthy group of piglets (Wilcoxon rank sum test, *p*<0.05); (B) Comparison of phylum level dominant groups and species level differences in intestinal fungi between the diarrhea group and the healthy group of piglets (Wilcoxon rank sum test, *p*<0.05). Phyla with relative abundance <1% are grouped into ‘Others’.

**Supplementary Results S2: Overall analytical workflow for microbiota-based diarrhea classification in neonatal piglets**

**
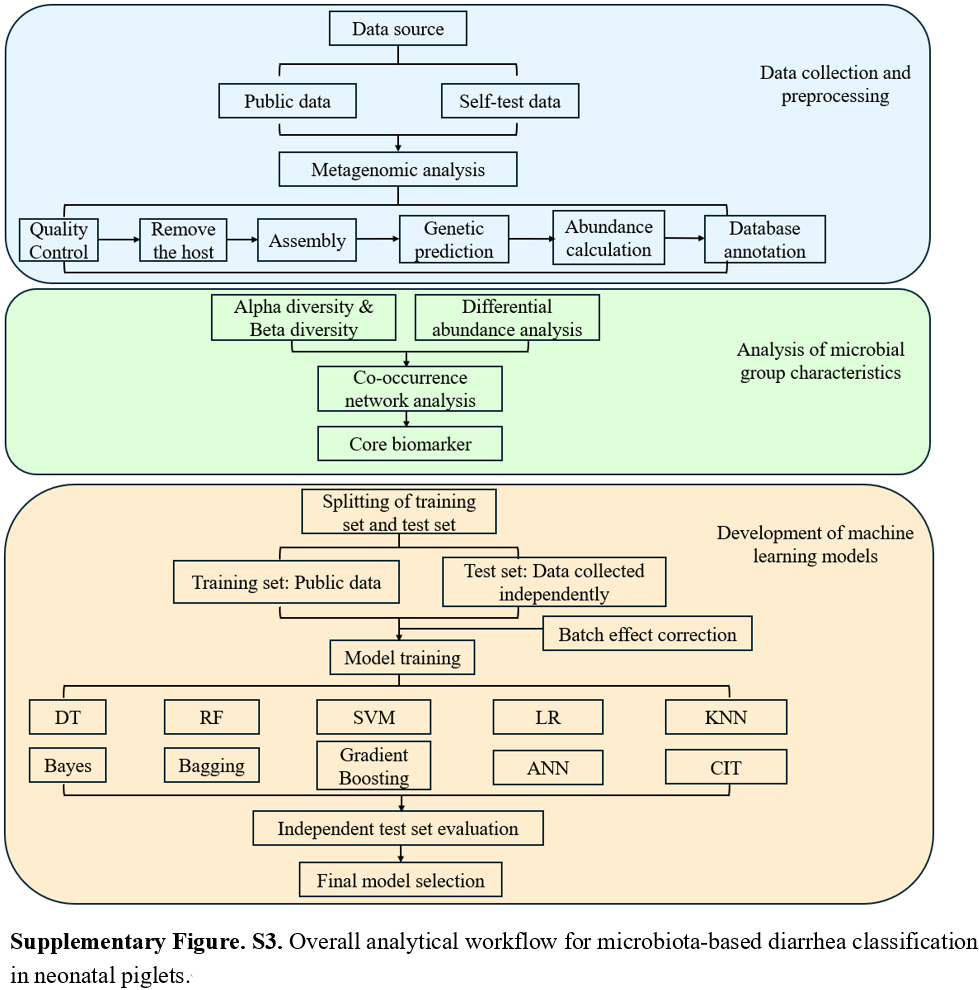
**

**Supplementary Figure. S3.** Overall analytical workflow for microbiota-based diarrhea classification in neonatal piglets. The workflow comprises five main stages: (1) data collection (public datasets and self‑collected samples); (2) metagenomic processing and microbiome characterization (α/β diversity, differential abundance, co‑occurrence network analysis); (3) core biomarker identification (18 core bacterial genera); (4) machine learning model training (10 algorithms, batch correction, cross‑validation); and (5) independent test set evaluation and final model selection.

**Supplementary Results S3: Comparison of the diarrhea index and bacterial F/B ratio for predicting piglet diarrhea**

After applying the feature selection criteria (described in Methods), 38 genera were identified as differentially abundant between diarrheic and healthy piglets (Supplementary Figure. S4).

To identify a minimal set of bacterial biomarkers with potential for future application via simpler techniques, the following analyses were conducted:

The Firmicutes-to-Bacteroidetes (F/B) ratio was calculated for each sample to assess its predictive power. The Wilcoxon rank-sum test revealed that the bacterial F/B ratio was significantly greater in diarrheic samples than in healthy samples (*p* < 0.05) (Supplementary Figure. S5A). A diarrhea index was calculated via the abundance information of bacterial phyla identified as significantly different (as described in the main text, Figure. 2). Specifically, it was defined as the sum of the relative abundances of phyla that significantly increased in diarrhea patients minus the sum of those that significantly decreased. The Wilcoxon rank-sum test revealed that the diarrhea index was significantly greater in diarrheic samples (*p* < 0.05) (Supplementary Figure. S5B). As both the diarrhea index and the F/B ratio are univariate, complex ML models were deemed unnecessary for their initial evaluation; thus, only the decision tree method was used for a preliminary comparison (Supplementary Figure. S5C).

(1) The prediction accuracy of the bacterial F/B ratio for piglet diarrhea was 61.76%, with an accuracy of 45.83% for diarrheic samples, 100% for healthy samples, and an AUC of 0.729.

(2) The prediction accuracy of the diarrhea index was 67.65%, with an accuracy of 54.17% for diarrheic samples, 100% for healthy samples, and an AUC of 0.629.

The results indicate that the overall predictive performance of the Diarrhea Index was slightly greater than that of the F/B ratio, but the accuracies of both indices were relatively low and substantially inferior to those of the ML models. Consequently, an artificial neural network (ANN), a "black-box" deep learning algorithm, was employed to model the F/B ratio and diarrhea index for predicting diarrhea, yielding the following results:

(1) When the ANN model was used, the prediction accuracy for the F/B ratio was 70.59%, with an accuracy of 58.33% for diarrheic samples, 100% for healthy samples, and an AUC of 0.792.

(2) When the ANN model was used, the prediction accuracy for the diarrhea index was 73.53%, with an accuracy of 70.83% for diarrheic samples, 80% for healthy samples, and an AUC of 0.754.
 The optimal predictive model for the F/B ratio was the ANN. Evidently, the ANN model significantly enhanced the predictive performance of both the diarrhea index and the F/B ratio, demonstrating its potential for further application.


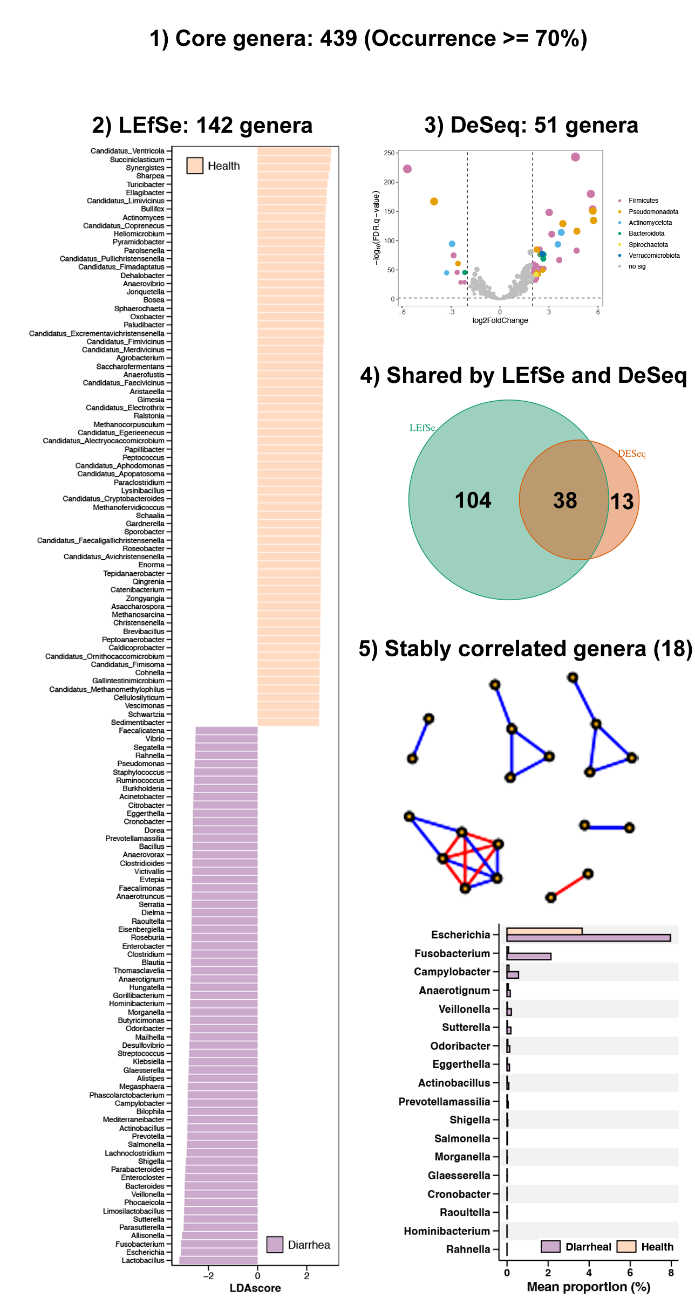


**Supplementary Figure. S4**. Analysis of bacterial biomarkers. Venn diagram showing the intersection of differential bacterial genera identified by LEfSe and DESeq2 analyses from 439 candidate genera prevalent in ≥70% of samples. A total of 38 common differential genera were obtained


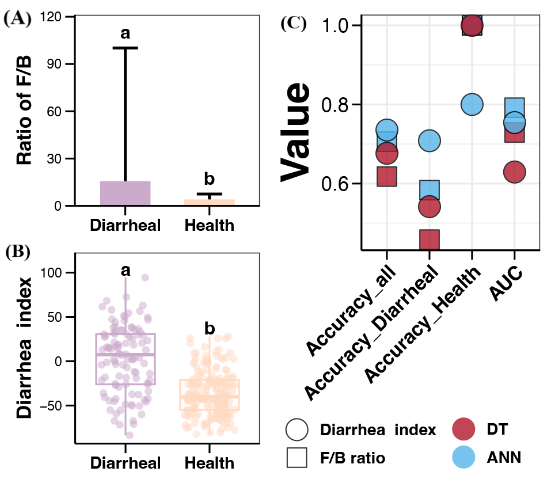


**Supplementary Figure. S5**. Comparison of the effects of the diarrhea index and bacterial F/B ratio in predicting diarrhea in piglets. (A) Boxplot of the F/B ratio; (B) Boxplot of the diarrhea index; (C) Performance metrics of decision tree models using the two indices.
